# Extended left hepatectomy for intrahepatic cholangiocarcinoma: hepatic vein reconstruction with in-situ hypothermic perfusion and extracorporeal membrane oxygenation

**DOI:** 10.1186/s12893-018-0342-2

**Published:** 2018-01-31

**Authors:** Deniz Balci, Menekse Ozcelik, Elvan Onur Kirimker, Arda Cetinkaya, Evren Ustuner, Mehmet Cakici, Bahadir Inan, Zekeriyya Alanoglu, Sadik Bilgic, Ahmet Ruchan Akar

**Affiliations:** 1 Department of General Surgery, Ankara University School of Medicine, K4 06590 Sihhiye, Ankara, Turkey; 2 Department of Anesthesiology, Ankara University School of Medicine, Ankara, Turkey; 3 Department of Radiology, Ankara University School of Medicine, Ankara, Turkey; 4 Department of Cardiovascular Surgery, Ankara University School of Medicine, Ankara, Turkey

**Keywords:** Intrahepatic cholangiocarcinoma, ECMO, Total vascular exclusion, In-situ hypothermic perfusion

## Abstract

**Background:**

Liver resection for intrahepatic cholangiocarcinoma (ICC) with invasion of the inferior vena cava (IVC) and hepatic veins (HV) is a challenging procedure.

**Case presentation:**

We report a case of a 63-year-old woman with a 6-cm, centrally located liver mass. Her biochemistry results were normal except for a Ca19–9 level of 1199 U/ml. The liver biopsy was consistent with ICC and 60% macrosteatosis. Abdominal CT scans revealed a large central mass invading the left HV, middle HV and right HV, infringing on their junction with the vena cava. An operation was planned using a 3-dimensional (3D) computer simulation model using dedicated software. We also describe a novel veno-portal-venous extracorporeal membrane oxygenation (VPV-ECMO) support with in-situ hypothermic perfusion (IHP) during this procedure. We aimed to perform an extended left hepatectomy and reconstruct 3 right HV orifices with an interposition jump graft to the IVC with total vascular exclusion (TVE) and IHP A supplemental video describing the preoperative planning, the operative procedure with the postoperative follow-up in detail is presented. After the patient was discharged, she developed a hepatic venous outflow obstruction 3 months postoperatively, which was effectively managed with hepatic venous stenting by interventional radiology. She is currently symptom free and without tumour recurrence at the 1-year follow-up.

**Conclusions:**

This report demonstrates that extended left hepatectomy for IHC with IHP and VPV-ECMO is safe and feasible under the supervision of a highly experienced team.

**Electronic supplementary material:**

The online version of this article (10.1186/s12893-018-0342-2) contains supplementary material, which is available to authorized users.

## Background

Liver resection offers curative treatment for intrahepatic cholangiocarcinoma (ICC), with 5-year survival reported between 30 and 50% after curative resection in both western and eastern populations [1]. Depending on tumour location and size, hepatic resection could be challenging, requiring thorough preoperative evaluation and planning. For complex liver resections, computer-assisted surgical planning using software for 3-dimensional (3D) reconstruction of the liver anatomy with better interpretation of a tumour’s relationship with critical vascular structures was reported to facilitate intraoperative decision-making with increased safety during resection [2]. In cases with invasion of the inferior vena cava (IVC) and hepatic veins where resection of these structures is required, additional safety measures, such as total vascular exclusion (TVE) to minimize bleeding, veno-venous bypass (VVB) for venous return and to prevent splanchnic congestion, and in-situ hypothermic perfusion (IHP) for liver preservation and complex reconstructions, may be required [3]. However, all these measures can potentially complicate the operation and the postoperative outcome. Normothermic liver ischaemia, especially for more than 60 min, and subsequent ischaemia-reperfusion injury elicit a risk of postoperative complications such as bleeding and liver failure (PLF) [4]. To decrease these risks, IHP with TVE is required so that the future liver remnant (FLR) can tolerate the ischaemia-reperfusion injury, which is critical for safe patient recovery [5]. Recently, the use of veno-venous extracorporeal membrane oxygenation (VV-ECMO) in the liver transplant setting was reported to be beneficial in facilitating operations in critical patients with complex comorbidities where prolonged cardiopulmonary support is required [6].

Here, we report a case of ICC with involvement of the IVC and hepatic veins. Using a 3D computer simulation model, we performed an extended left hepatectomy and reconstruction of 3 right hepatic veins under IHP and veno-portal-venous extracorporeal membrane oxygenation (VPV-ECMO) support.

## Case presentation

A 63-year-old obese, diabetic woman with a history of previous cholecystectomy was admitted to our institution with right upper quadrant pain. Her body mass index was 36. She was positive for Factor 5 Leiden heterozygous mutation. Abdominal Ultrasonography (USG) revealed a centrally located 6-cm mass with grade 3 hepatosteatosis. Fine needle biopsy was consistent with adenocarcinoma of biliary origin, which was interpreted as intrahepatic cholangiocarcinoma (ICC). Abdominal computed tomography (CT) scans revealed a large central mass invading the left (LHV), middle (MHV) and right hepatic veins (RHV) infringing upon their junction with the vena cava. An additional movie file shows this in more detail (see Additional file 1: Video S1). Her biochemistry results were normal other than a Ca19–9 level of 1199 U/ml. Positron Emission Tomography confirmed liver-only disease and a single liver mass with SUV Max: 7.5.**Additional file 1: Video S1.** This video consists of surgical procedure and postoperative course. (MP4 275,443 kb)

### Operation plan

The operation was planned using computer assisted 3-dimensional (3D) modelling software for 3D reconstruction of the liver scan images (Myrian, Intrasense France). The IVC and the RHV, MHV and LHV junction were affected by the tumour. Therefore, resection and reconstruction of these structures were planned. The resection plane was modified to obtain the maximum volume of the FLR, including segments 5–6-7 of the right lobe (61% of total liver volume), because of the patient’s age and the degree of hepatosteatosis. It was estimated that the critical part of the parenchymal transection would be providing a safety margin while following an initially anatomical and then non-anatomical resection plane to find the junction where the RHV separated into 3 branches before invasion by the tumour. We planned to resect and reconstruct the IVC followed by a jump graft reconstruction of the 3-orifice junction to the IVC. We intended to perform the critical transection and reconstruction steps using TVE and VPV-ECMO with IHP for FLR protection. The submitted multimedia (see Additional file 1: Video S1) shows the operative plan with 3D reconstruction.

The patient was taken to the OR, but during the initial phase of anaesthesia induction, she experienced an anaphylactic reaction in the form of severe bronchoconstriction associated with increased airway pressure, profound hypoxaemia and hypercarbia. Despite all efforts with conventional medical treatment for severe bronchoconstriction and lung protective ventilation strategies, the patient did not recover until reversal of the neuromuscular blocker rocuronium with sugammadex, a reversal agent for rocuronium. The operation was cancelled to allow the patient to recover and detailed allergy tests were conducted. After comprehensive studies with the objective of determining safe anaesthetic agents, including neuromuscular blockers, the operation was performed 2 weeks later. Anaesthetic induction was maintained with thiopental, vecuronium, fentanyl and midazolam without any adverse events. The right femoral artery was cannulated with a 5 Fr central venous cannula for advanced haemodynamic monitoring of continuous cardiac output with pulse contour analysis (FloTrac system, software version 1.01; Edwards Lifesciences, Irvine, CA, USA). To derive more detailed haemodynamic data, including tissue oxygenation, an 8 Fr pulmonary artery catheter (CCombo, Edwards Lifesciences, Irvine, CA, USA) was inserted into the left internal jugular vein.

We planned to use VPV-ECMO rather than standard VV-Bypass to provide not only perfusion but also support for oxygenation and respiratory function and to avoid splanchnic congestion during the course of the procedure.

### The VPV-ECMO setup

The ECMO circuit used in this case was a continuous flow centrifugal pump (JostraRotaflow®; Maquet Cardiopulmonary, Rastatt, Germany), oxygenator (JostraQuadrox®; Maquet Cardiopulmonary, Rastatt, Germany), and heat exchanger. After heparin administration (5000 IU), USG-guided right common femoral vein (RCFV) puncture was performed. A flexible J-tip guide-wire was advanced through the RCFV. Using the Seldinger technique, a 21 Fr multi-perforated venous cannula (HLS®Cannulae, Maquet®, Rastatt, Germany) was inserted over the guide-wire up to the infra-hepatic IVC. The position was confirmed with manual palpation of the catheter tip within the IVC, located just below the level of the left renal vein. Another 17 Fr cannula (Arterial ®Cannulae, Maquet®, Rastatt, Germany) was inserted over a guide-wire through the right internal jugular vein (RIJV) down to the right atrium under transesophageal echocardiography guidance. Furthermore, a 16 Fr venous cannula (Edwards Lifescience, TF016L, LLC, Irvine, CA, USA) was inserted to the portal vein (PV). The RCFV and PV cannulas served as drainage cannulas and the RIJV cannula served as a supplying cannula for the VPV-ECMO set-up. Using flow sensors, we monitored flow ratios in the draining cannulas during the procedure; maximum VPV-ECMO flow was 3.2 L/min and maximum PV flow was 0.5 L/min. PO_2_, PCO_2_, SO_2_, pH and lactate levels were monitored via right radial arterial blood samples. Maximum serum lactate was 2.75 mmol/Lt during the procedure.

### The operation

A median laparotomy was performed. The operation started with TVE of the liver for inflow and outflow control (see Additional file 1: Video S1). An intraoperative USG examination was performed by an experienced radiologist (EU) to confirm the tumour’s relationship with vascular structures and the anticipated tumour-free FLR. The hepatoduodenal ligament was dissected, isolating the main (MPV), right (RPV) and left portal (LPV) veins, followed by left (LHA) and right hepatic artery (RHA) dissection.

Hepatoduodenal ligament dissection was completed after a 16 Fr IHP cannula was inserted from the LPV. Selective control of the left hilar pedicle by tying both the LHA and LPV revealed a demarcation line on the liver and parenchymal transection proceeded using an ultrasonic surgical aspirator (Sonastar, Misonix USA) following the MHV until the point of preoperatively planned deviation using the 3D model from the anatomical line (MHV) was reached. At this point, although we had planned to resect the IVC with the tumour, we were able to dissect and preserve it using the ultrasonic surgical aspirator. In this step, we obtained three frozen biopsies from the tissue showing a desmoplastic reaction between the IVC and liver capsula, demonstrating an invasion-free IVC.

IHP was initiated before the LHV, MHV and RHV were cut and the liver was elevated through the incision for the final critical step of parenchymal transection. The 3 right hepatic vein branches were exposed (see Additional file 1: Video S1). After the hepatectomy was completed, an 8-cm interposition cadaveric IVC graft was used for reconstruction of the three hepatic vein orifices, joining them with the patient’s preserved IVC. We first anastomosed the right hepatic vein junction with the IVC graft that matched the total diameter of the 3 RHV orifices using 4.0 prolene in an end-to-end fashion. Then, we anastomosed the graft to the RHV orifice on the IVC that was enlarged in both inferior and medial directions, finally matching the diameter of the graft. After the reconstruction was complete, we terminated IHP and removed the ECMO cannula in the MPV. During and immediately after reperfusion, we kept the femoral vein cannula in place and the ECMO system running. The RCFV and RIJV cannulae were removed after haemostasis and confirmation of haemodynamic and respiratory stability.

The total duration of the operation was 12 h. Four units of erythrocytes and 3 l of HTK solution were required for IHP, which was performed for a total of 82 min.

The patient was discharged from the ICU 48 h after the operation with gradual improvement of liver function. Pathology confirmed a 6-cm, grade 2 adenocarcinoma of biliary origin. The tumour showed lymphovascular invasion. Three hepatoduodenal lymph nodes were negative and the operation was considered an R0 resection with a less than 1-mm surgical margin in the parenchymal transection line close to the hepatic veins.

On postoperative day 5, despite anticoagulation prophylaxis, the patient experienced pulmonary embolism, which caused haemodynamic instability and required intubation, mechanical ventilation, and inotropic support. She was readmitted to the ICU. However, there was no evidence of myocardial injury as indicated by a negative cardiac troponin test. Unfractionated heparin was administered as an intravenous bolus followed by a therapeutic dose of low-molecular weight heparin. After a gradual recovery, she was ambulated and discharged after 32 days of hospitalization.

At 3 months, she experienced abdominal pain, ascites and jaundice. A triphasic CT scan showed outflow obstruction with kinking of the venous graft due to extensive liver regeneration. The patient’s condition deteriorated, with an increase in total bilirubin to 9 mg/dl and massive ascites and hydrothorax. The patient was taken to an angiography suite where a metallic stent was inserted, resulting in rapid recovery of liver function and the patient’s condition (see Additional file 1: Video S1).

At the 1-year follow up, she is symptom-free. Her Ca19–9 level is 35 U/ml. Her follow-up CT scan shows no tumour recurrence and normal liver function tests.

## Discussion and conclusions

Over the last 2 decades, advances in surgical techniques, perioperative care and interdisciplinary teamwork represent a paradigm shift for patients undergoing complex liver reconstruction with vascular resection. In recent years, technological advances have been established for preoperative planning and perioperative support, which may affect operative mortality, morbidity, and long-term outcomes of these patients in experienced centres [5, 7].

In complex liver resections requiring HV-IVC reconstruction, TVE becomes an important safety measure when bleeding is anticipated, but haemodynamic instability with splanchnic venous congestion causes ischaemia and eventually reperfusion injury (IRI) after de-clamping. Hepatic IRI, an inflammatory response that follows hepatic ischaemia and is characterized by overproduction of reactive oxygen species (ROS), has detrimental effects on FLR recovery after extended liver resections. To avoid this, IHP has been reported to be a protective manoeuvre that may potentially increase the liver’s tolerance to ischaemia and may be helpful in the recovery of hepatic function and regeneration in the normal liver, but it has not been thoroughly studied in the context of damaged liver parenchyma [8]. The patient in the case reported here was relatively older with a significant degree of parenchymal steatosis (60% macrovesicular), which are both reported significant risk factors for severe morbidity and mortality after major liver resection [9]. To optimize FLR recovery, parenchymal preservation with IHP and the use of TVE with VPV-ECMO provide additional safety measures for patients with significant comorbidities. To our best knowledge, this is the first report of VPV-ECMO support in extended left hepatectomy, which was designed and configured by our multidisciplinary team (ARA, DB, MO). Recently, we reported the effective use of VPV-ECMO support in critically ill liver transplant recipients during IVC resection and reconstruction in the LDLT setting [10]. There are several advantages of VPV-ECMO support during hepatectomy over conventional techniques. In brief, VPV-ECMO 1) drains portal circulation and mitigates splanchnic congestion and edema, 2) provides partial or complete support for oxygenation and respiratory function, 3) allows ventilation with lower tidal volumes and lower pressures, and 4) provides better myocardial oxygenation and 5) pulmonary vasodilation. Furthermore, VV-ECMO support can be continued after surgery if required. However, the technique requires expertise and has several disadvantages, including an increased risk of bleeding during surgery because of moderate anticoagulation during support, a potential inflammatory response to extracorporeal circulation, and a risk of vascular complications.

For preoperative planning of complex liver resections, 3D reconstructions with computer simulation models are showing broader clinical application for volumetric calculations and parenchymal transection plane determination [11]. We used a similar approach to choose the FLR and determine the parenchymal transection plane and for planning the outflow reconstruction with 3 RHV junctions to the IVC. Although outflow reconstruction using a native IVC graft enabled successful recovery of the patient in the early period, after 3 months, graft regeneration in the anterior-posterior and lateral to medial directions resulted in graft tension and kinking, with symptoms of outflow obstruction requiring endovascular intervention. Outflow reconstruction problems have been studied in detail in the living-donor liver transplantation (LDLT) setting and the general solution is to create larger openings with consideration of graft regeneration patterns [12]. However, in LDLT, the distance between the graft RHV and the recipient IVC is much shorter than an atypical resection as presented here that created a longer gap. Alternatively, a ringed Gore-tex or a dacron graft could be a better choice than a native graft due to better patency, but timely diagnosis and endovascular hepatic venous stenting is helpful in this clinical scenario [13].

This report demonstrates that complex liver resection with vascular reconstruction is safe and feasible with preoperative planning using 3D computer simulation, intraoperative VPV-ECMO for cardiorespiratory support and a close postoperative follow-up using a multidisciplinary team approach. Using a combination of techniques described could potentially increase the number of patients with centrally located liver tumors of either metastatic or primary disease (ie. cholangiocellular carcinoma) with various degree of liver parenchymal quality and function who could safely undergo complex liver surgeries that is otherwise unresectable.

## Abbreviations

3D: 3-dimensional
CT: Computed tomography
FLR: Future liver remnant
ICC: Intrahepatic cholangiocarcinoma
IHP: In-situ hypothermic perfusion
IVC: Inferior vena cava
PV: Portal vein
RCFV: Right common femoral vein
RIJV: Right internal jugular vein
TVE: Total vascular exclusion
VPV-ECMO: Veno-portal-venous extracorporeal membrane oxygenation
VVB: Veno-venous bypass
